# Kidney Organoids as Disease Models: Strengths, Weaknesses and Perspectives

**DOI:** 10.3389/fphys.2020.563981

**Published:** 2020-11-04

**Authors:** Ricardo Romero-Guevara, Adonis Ioannides, Christodoulos Xinaris

**Affiliations:** ^1^University of Nicosia Medical School, Nicosia, Cyprus; ^2^Istituto di Ricerche Farmacologiche Mario Negri IRCCS, Centro Anna Maria Astori, Science and Technology Park Kilometro Rosso, Bergamo, Italy

**Keywords:** stem cells, kidney, organoid, 3D cell culture, hiPSC, disease modeling

## Abstract

Chronic kidney disease is a major global health problem, as it affects 10% of the global population and kills millions of patients every year. It is therefore of the utmost importance to develop models that can help us to understand the pathogenesis of CKD and improve our therapeutic strategies. The discovery of human induced pluripotent stem cells (hiPSCs) and, more recently, the development of methods for the generation of 3D organoids, have opened the way for modeling human kidney development and disease *in vitro*, and testing new drugs directly on human tissue. In this review we will discuss the most recent advances in the field of kidney organoids for modeling disease, as well as the prospective applications of these models for drug screening. We will also emphasize the impact of CRISPR/cas9 genome engineering on the field, point out the current limitations of the existing organoid technologies, and discuss a set of technical developments that may help to overcome limitations and facilitate the incorporation of these exciting tools into basic biomedical research.

## Introduction

Chronic Kidney Disease (CKD) is causing an emerging global healthcare crisis (Couser et al., 2011)^.^ 10% of the population worldwide is affected by this disease, and millions die each year because they do not have access to affordable treatment. CKD often leads to end stage renal disease (ESRD), for which patients require either hemodialysis or kidney transplantation in order to survive. However, both renal replacement therapies are insufficient: dialysis substitutes only a small percentage of renal function and does not correct the compromised endocrine functions of the kidney, while the usefulness of transplantation is limited by the shortage of donor organs and the subsequent need for lifelong immunosuppressive therapy. Considering the globally increasing prevalence and annual incidence of CKD, more efficient therapeutic options are urgently needed.

Being able to engineer human organoids in a dish would significantly improve our ability to cure and manage kidney diseases, and fundamentally change the way we conduct biomedical research. First, it would allow scientists to study the disease and explore new therapies directly on human tissue, which would significantly improve the translatability of candidate drugs. Second, organoids could be used to model normal human development and diseases in a personalized manner. Finally and most importantly, organoids could be used in replacement therapies, which would make possible the unlimited production of transplantable tissues and would solve the problem of organ shortages once and for all. However, how far organoids are from fulfilling these expectations remains unclear because of significant technical limitations.

In this paper we will provide an overview of the current progress in the field of kidney organoids, with specific emphasis on the use of organoids for disease modeling and drug testing. We will also discuss how the current organoid technologies can be exploited, analyze the existing limitations, and propose technical improvements that may help to overcome limitations and ultimately facilitate the incorporation of these exciting tools in biomedical research.

## The Strengths of Kidney Organoids

**FIGURE 1:**
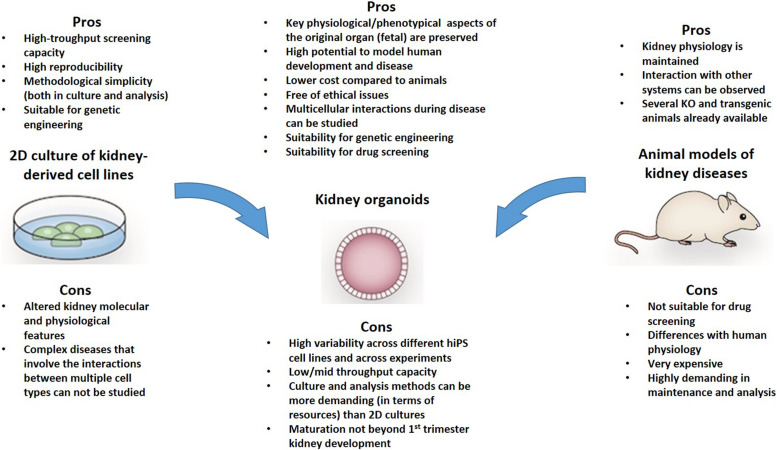
The Pros and cons of kidney organoids for modeling human disease are highlighted in this figure. It is possible that some of the limitations of this system may be overcome with modern technics for miniaturization and culture in microfluidic devices that allow a physiological assessment of podocytes and tubular cells.

Organoids have significant advantages compared with animal models and traditional 2D cultures. First, compared with animal models, organoids allow more opportunities for experimental manipulations, as they are isolated multicellular systems, are amenable to real-time imaging techniques, and, most importantly, enable the study of human developmental processes and pathogenetic pathways that are not easily or accurately replicated in animal models (Figure 1). Compared with monolayer traditional cultures, organoids contain more than one cell type. This enables more “physiological” modeling, as they can replicate various aspects of the disease, especially when the pathogenesis involves interactions between different cell types – as commonly occurs in kidney diseases. Normal renal function depends not only on cellular homeostasis, but also critically depends on the architecture of both the individual cells and the organ. The nephrons (the filtering units of the kidney) work through a complex multi-step process, which involves a specialized microvascular bed (containing fenestrated endothelial cells, highly specialized podocytes, and mesangial cells) that produces the primary filtrate, and an epithelial tubule that returns needed substances to the blood and pulls out additional waste. Studying the mechanisms (or even only some aspects of the mechanisms) governing these processes would require multicellular systems with a high degree of organization. For instance, classic 2D cultures could not model certain glomerulopathies as efficiently as organoids because of the multifactorial etiopathogenesis of the disease, which involves interactions between these different cell types and alterations in tissue organization. Thus, it is sound to assume that the more an experimental model replicates the above processes, the more efficient it would be for modeling human diseases and more reliable mechanistic studies. Therefore in view of the complexity of the kidney, organoids are the most complete *in vitro* experimental model that is currently available.

Importantly, organoid cells maintain genome stability and phenotype better compared with primary cultures, which makes them suitable for biobanking and high-throughput screens. Organoids display a higher degree of organization, specialization and maturation compared to their 2D culture counterparts. For instance, it has been shown that tubular cells in organoids mature sufficiently to uptake fluorescent dextrans from the lumen of tubules (Freedman et al., 2015; Takasato et al., 2015; Li et al., 2016), in contrast with the loss of cellular polarity and low or absent expression of drug transporters observed in immortalized kidney tubular cell lines (Jenkinson et al., 2012; Nieskens and Sjogren, 2019). In fact, a comparison of primary cultures of proximal tubular epithelial cells, immortalized kidney cell lines and renal tissue showed that several enzymes and receptors that are involved in the xenobiotic metabolism are missing from cultured cells (Van Der Hauwaert et al., 2014). The expression of these metabolic genes plays an important role in tubular function and nephrotoxicity, and this lessens the applicability and robustness of cell culture models in toxicity screens. Moreover, primary cultures of tubular cells exhibited limited proliferation capacity, while the expression of drug metabolism genes decreases quickly during culture (Lash et al., 2008), limiting their application in drug screening.

Significant advances have also been made in attempts to generate glomeruli from hiPSCs. Several studies have shown that podocytes in organoids are organized in glomerular structures containing endothelial cells, and nephrin-positive podocytes with immature foot process and apico-basal polarity of tight junction proteins (Kim et al., 2017; Yoshimura et al., 2019), as well as having a basic filtering capacity when transplanted *in vivo* (Van Den Berg et al., 2018). Another important feature of organoids – that is particularly useful for toxicity studies and disease modeling – is their ability to respond to stress by expressing and/or releasing injury markers in specific cell types, as observed in kidney tissue. For example, in hiPSC-organoids treated with gentamycin or cisplatin, KIM1 was specifically expressed in tubular epithelial cells (Freedman et al., 2015; Morizane et al., 2015). Interestingly, LTL + cells also expressed cleaved caspase 3 (CASP3) following cisplatin treatment, and this effect was only observable in proximal tubular cells of day 18 organoids and not at earlier time points or in other cell types (Takasato et al., 2015). Altogether, these features highlight the higher level of maturity of organoids vs. traditional 2D culture models, which can be exploited for disease modeling, drug screening and toxicological studies.

Nowadays, there are several protocols for generating kidney organoids using embryonic stem cells (ESCs) or hiPSCs that can be derived from patients or healthy donors. These protocols include one or more steps of Wnt activation (by CHIR99021) and a cocktail of secreted factors that can yield organoids with various kidney structures and cell types (Table 1; Taguchi et al., 2014; Taguchi and Nishinakamura, 2017; Howden et al., 2019).

**TABLE 1:** Main protocols for generating kidney organoids from human pluripotent stem cells: differentiation stages and times, and characterization of final observed cells.

Kidney organoid differentiation protocols			
References	Differentiation stages	Length	Cells obtained
Melissa H. Little group Takasato et al., 2014; Takasato et al., 2015; Forbes et al., 2018; Howden et al., 2019	Induction of primitive streak through CHIR99021, followed by FGF9 treatment to induce intermediate mesoderm and MM, plus 3D culture for generating organoids.	18–24 days	Proximal and distal tubule cells, podocytes, and collecting duct cells.
Nishinakamura group Taguchi et al., 2014; Taguchi and Nishinakamura, 2017; Yoshimura et al., 2019	The protocol starts with the formation of embryoid bodies in the presence of BMP4, followed by early mesoderm induction with Activin and Fgf2, and then a cocktail of BMP4, CHIR99021, Retinoic acid to induce intermediate mesoderm and MM. NPCs were then expanded with Fgf9 and CHR99021. For nephron maturation, 3D aggregation and coculture with mouse embryonic spinal cord was used. For inducing the ureteric bud specifically, after the initial mesoderm induction cells were treated with blockers of BMP/Activin signaling LDN193189 and SB43152. In both protocols UB or MM progenitors are sorted for further maturation in 3D culture.	22 days	Glomeruli with podocytes, proximal and distal tubule cells. In Taguchi and Nishinakamura, 2017, UB-derived collecting duct cells were generated.
Izpisua-Belmonte group Xia et al., 2014; Li et al., 2016; Low et al., 2019	These protocols are composed of an initial stage in monolayer with intermittent CHIR99021 treatment to induce intermediate mesoderm, followed by Fgf9 and CHIR99021 for NPCs induction. Then in a second stage, 3D culture of sorted SIX2 + NPCs with Fgf9 and CHIR is maintained, followed by basal media without growth factors. Ureteric bud differentiation is induced by monolayer culture in Fgf2 and Bmp4, followed by Bmp2, Activin A and retinoic acid.	21 days	Glomeruli with podocytes, proximal and distal tubule cells, as well as endothelial cells.
Bonventre and Freedman group Freedman et al., 2015; Morizane et al., 2015; Morizane and Bonventre, 2017	Primitive streak is induced by CHIR treatment in monolayer culture. Then Activin is added to induce intermediate mesoderm, and NPCs are expanded at the presence of Fgf9. Finally cells are aggregated and cultured for 3 days at the presence of Fgf9 and CHIR99021, and then switched to basic media without growth factors and inhibitors.	28–35 days	Podocytes, proximal and distal tubule like cells were observed.
Przepiorski et al., 2018	This protocol is based on the formation and growth of embryoid bodies from hiPSCs, in a bioreactor culture system, The only supplemented used is CHIR99021. The expansion of NPCs within the embryoid bodies is achieved with Knockout serum replacement media instead of Fgf9 as the majority of the differentiation protocols.	14–26 days	Podocytes, proximal and distal tubules, and stromal cells.

Alternatively organoids can also be derived from embryonic kidney progenitors (Xinaris et al., 2012) or adult tissue cells (Schutgens et al., 2019). Interestingly, the recently developed protocols for the generation of tubuloids from Wilms tumors allowed for the establishment of an organoid biobank for childhood kidney cancers and identifying patient-specific drug sensitivities (Calandrini et al., 2020). In addition, with the emergence of genome engineering technologies such as CRISPR/Cas9, hiPSCs can be modified to introduce disease-specific mutations, correct them, or introduce genetic reporters that are useful for drug screening. Given all these achievements, it is reasonable to ask how we can we use kidney organoids in routine biomedical research.

## Current Applications

The nearest use of kidney organoids in biomedical research is for studying the mechanisms of kidney diseases, drug discovery and toxicological studies. Despite the great interest in using organoids for disease modeling, so far few human diseases have been successfully modeled in kidney organoids. Nevertheless, the examples described below suffice to provide concrete proof-of-concept for the potential of kidney organoids in biomedical research.

### Disease Modeling

#### Ciliopathies

In one of the earliest examples of using organoids to model polycystic kidney disease (PKD), Freedman et al. (2015) knocked out the *PKD1* and *PKD2* genes in hiPSCs using CRISPR/Cas9 genome editing technology and used these cells to construct kidney organoids. After 35 days in culture, cysts spontaneously formed within the tubules of biallelic *PKD1*-/-, *PKD2*-/- hiPSCs-derived organoids but not in isogenic controls, validating the organoid culture to model PKD. One limitation of this study was the low frequency of cystogenesis (only 6% of organoids developed cysts). In a follow-up study, cystogenesis was improved (up to 75% of organoids developed cysts) by growing organoids in suspension culture compared to adherent conditions (Cruz et al., 2017). Organoids may also help unravel the complex molecular and cellular events that underlie the pathogenesis of PKD and, in doing so, help identify novel targets for disease modulation and therapy. The same group adapted this platform to identify modifiers of PKD, and showed that the non-muscle myosin II inhibitor blebbistatin promotes cystogenesis in this disease model, potentially implicating the myosin pathway and the regulation of actin-myosin activation in these conditions (Czerniecki et al., 2018). In many cells, non-muscle myosin has been involved in adhesion, cell polarity, migration and endocytosis and exocytosis but its specific function in tubular cells has not been elucidated yet (Noris and Remuzzi, 2012). Thus, this study is a paradigm of how these tools can be used to investigate novel aspects of the pathogenesis of as complex a genetic disease as PKD (Czerniecki et al., 2018).

Although gene-edited knockout (KO) hiPSCs exhibited a remarkable ability to form cysts, PKD patient-derived hiPSCs exhibited dramatic line-to-line variability in their abilities to form organoids containing cysts, regardless of PKD genotype and organoid differentiation protocol (Cruz et al., 2017). The morphology of tubular structures also varied noticeably between different lines. Nonetheless in a recent publication, the same group also generated hiPSCs from a patient affected by autosomal recessive PKD (ARPKD). As with PKD KO cells, the ARPKD patient-derived organoids also developed cysts upon exposure to cAMP agonists (Low et al., 2019), proving the concept for modeling PKD in the same genotype as the patient. It remains to be seen if such a culture methods can also efficiently induce cystogenesis in other genotypes from different patients.

Establishing robust protocols for the generation of kidney organoids from hiPSCs of patients with Autosomal Dominant PKD (ADPKD) could create an excellent opportunity to study genotype-phenotype correlation. The majority of cases of ADPKD are caused by pathogenic mutations in the *PKD1* gene and these patients have, on average, an earlier age at diagnosis and onset of ESRD than patients with *PKD2*-associated ADPKD. It is worth noting that there is variation in disease severity amongst patients with different *PKD1* mutations. In keeping with their loss-of-function effect, truncating mutations cause more severe disease compared to non-truncating ones and there is variation within the latter group, depending on the impact on protein structure and function (Heyer et al., 2016; Hwang et al., 2016). More severe disease, including embryonic lethality, intrauterine onset of cystogenesis and diagnosis in childhood, can be caused by biallelic mutations in either the *PKD1* or *PKD2* genes. Personalized genotype-specific organoids will be of crucial significance for studying the molecular mechanisms that govern the different clinical phenotypes, and developing self-tailored approaches to disease monitoring and management.

Other mutations that are associated with the formation of renal cysts have also been studied in hiPSC-derived kidney organoids. Przepiorski and colleagues generated hiPSCs-derived kidney organoids with point mutations to knockout *HNF1B* that could phenocopy nephron defects observed in *Hnf1b* conditionally deficient mice. Nevertheless, these organoids did not develop renal cysts (as happen in *Hnf1b*^–/–^ mice and individuals with heterozygous mutations in *HNF1B*) highlighting the key phenotypical and mechanistic differences between organoid models and the *in vivo* patho-biology of the disease (Przepiorski et al., 2018).

Another recent example in which a congenital disease has been modeled in organoids is a case of IFT140 nephronophthisis-related ciliopathy (Forbes et al., 2018). In this study, the authors generated hiPSC-derived organoids from a patient with *IFT140* mutations and isogenic mutation-corrected hiPSCs, and showed that *IFT140* mutations can cause defects in primary cilia and alter apico-basal polarity in tubular cells. Interestingly, these alterations were consistent with ciliary defects observed earlier in *Ift140* knockout mouse (Miller et al., 2013).

#### Mucin 1 Kidney Disease

Proteinopathies are caused by the intracellular accumulation of misfolded proteins, activating several stress response pathways, such as unfolded protein response (UPR), endoplasmic reticulum stress and ultimately cell death. One of such pathologies, mucin1 kidney disease (MKD), is the result of a frame shift mutation in the *MUC1* gene (*MUC1-fs*), which introduces a premature stop codon and leads to the synthesis of a shortened mutant protein that is accumulated in the cytoplasm (Kirby et al., 2013). Greka’s lab recently used complementary organoid and *in vivo* models to study this proteinopathy, and demonstrated an intracellular accumulation of mutant MUC1 transmembrane protein in tubular cells of mutant transgenic mice, as well as in human MKD samples and hiPSC-derived organoids from patients (Dvela-Levitt et al., 2019). In addition, the effectiveness of the BRD4780 compound, selected from a primary screen using mutant *MUC1-fs* immortalized tubular epithelial cells, was demonstrated in the three experimental models by targeting mutant protein to lysosomal degradation.

#### Podocytopathies

Another important milestone has been the use of patient-derived organoids to study the pathogenesis of congenital nephrotic syndrome. In the Tanigawa et al. (2018), hiPSC-derived organoids from a patient with nephrotic syndrome caused by *NPHS1* mutations were transplanted under the kidney capsules of immunodeficient mice and used to study and identify slit diaphragm abnormalities in podocytes. Moreover, the use of CRISPR/cas9 gene editing technology to correct the *NPHS1* mutation restored the podocyte transcriptional profile and rescued the disease phenotype. hiPSC-derived organoids have also been used to model *NPHS1*-related nephrotic syndrome in the study by Hale et al. (2018) and revealed hyperthrophied podocytes cell bodies and reduced levels of the podocytes proteins nephrin and podocin (Hale et al., 2018).

hiPSC-derived organoids have also been used to study the role of podocalyxin in kidney development (Freedman et al., 2015; Kim et al., 2017). Podocalyxin is important for maintaining the foot process, and knockout mice die soon after birth (Doyonnas et al., 2001). In human organoids, it was shown that podocalyxin plays an important role in providing a negative charge to the cell surface of podocytes that is necessary for epithelial lumen formation and the organization of the podocyte’s tight junctions, and it is necessary for microvilli formation (Freedman et al., 2015; Kim et al., 2017). Interestingly, key structural alterations in podocalyxin-deficient mice are partially phenocopied in human kidney organoids with defective podocalyxin, stressing the similarity between organoids and *in vivo* system. It should be pointed out, however, that null mutations in the *PODXL* gene have not been definitively associated with human kidney disease, though a missense variant of unknown significance that affects the podocalyxin transmembrane domain has been identified in a family with focal and segmental glomerulosclerosis (Barua et al., 2014). Still, existing data suggest that kidney organoids can be used to study the role of podocalyxin in normal organogenesis and *PODXL*-associated developmental defects. A summary of the human diseases modeled in organoids is presented in Table 2.

**TABLE 2:** The main outcomes, limitations and future perspectives of the hiPSCs-derived organoids models of kidney diseases are described.

Kidney organoid models of disease			
Model-disease type and references	Outcomes	Limitations and challenges	Future perspectives
Mucin 1 kidney disease (MKD) Dvela-Levitt et al., 2019	Human iPSC-derived organoids from MKD patients exhibited Muc1 protein mislocalization in tubular cells. Organoid cells responded to the drug BRD90 in a similar manner as in mouse models and patients with MKD.	No functional assays, such as tubular absorption, were carried out in MKD organoids.	Developing semi-automated culture systems and analysis approaches can make this system a valuable drug testing tool for MKD.
Polycystic kidney disease (PKD) Freedman et al., 2015; Cruz et al., 2017; Czerniecki et al., 2018; Low et al., 2019	These human organoids from PKD1/PKD2 KO hiPSCs extensively formed cysts *in vitro* and can be adapted to high-throughput screening. Using this culture format, a potential involvement of non-muscle myosin in cystogenesis was identified.	PKD patient-derived hiPSCs did not sufficiently form cysts, hampering the application of this organoid system to study the genotypes of patients.	Optimizing culture conditions to induce cysts in patient-derived organoids will provide a highly useful assay for studying disease pathogenesis and developing personalized therapeutic strategies.
ITF40 Nephronophthisis ciliopathic renal disease Forbes et al., 2018	Somatic cells from a patient with compound heterozygous mutations in *IFT140* were reprogrammed and corrected with CRISPR/Cas9. Primary cilia and apico-basal defects were observed in tubular cells of patient-derived kidney organoids but not in gene-corrected isogenic controls.	Primary cilia and spheroid polarity defects were not present in all organoids, which limits the potential use of the system in drug screening applications.	Generation of iPSC-derived organoids from patients with different mutations in NPHP genes will help to elucidate the mechanisms of Nephronophthisis pathogenesis and explain the differences between the clinical phenotypes.
Podocalyxin deficiency Kim et al., 2017	Knockout of podocalyxin demonstrated its importance for microvilli formation and podocyte spacing. Podocalyxin-KO organoids phenocopied, to some degree, the pathological features of the kidney in podocalyxin knockout mice, validating the use of organoids for understanding human podocyte development.	Podocalyxin deficiency is not compatible with life, and newborns with such defects are rarely reported. Therefore, although podocalyxin-deficient organoids can be used to study the role of podocalyxin in early steps of organogenesis, are less useful for studies in adult human diseases.	A mutation variant of unknown significance in *PODXL* has been identified in humans, the generation of patient-specific hiPSCs and their differentiation could be a valuable tool to investigate the pathogenesis of *PODXL* mutations in humans.

### Nephrotoxicity Testing

Kidney organoids can potentially be used in toxicological studies as well (Table 3). Unlike classic kidney cell lines, organoids can provide a platform for evaluating the different responses of the various cell types simultaneously and following secondary intercellular responses after nephrotoxic injuries. In addition, the higher level of maturity of organoids compared to immortalized cell lines, allow us to obtain more physiologically relevant data. For example, nephrotoxic drugs such as cisplatin, which mainly target the proximal tubules *in vivo*, have also shown specific toxicity for proximal tubular cells in organoids (Freedman et al., 2015; Morizane et al., 2015). Moreover, tubular toxicity was stronger in organoids at day 18 than in less mature organoids on day 11 (Takasato et al., 2015). These data suggest that organoids could be used to study the differential effect of nephrotoxic drugs on renal cells on the basis of their phenotypical (functional and structural) characteristics and differentiation state.

**TABLE 3:** The proof-of-concept studies in which hiPSCs-derived kidney organoids have been developed as potential models for nephrotoxicity and drug screening are presented, highlighting the main outcomes, limitations and future areas of improvement for these models.

Drug screening and toxicity assays applications of kidney organoids			
Organoid application/references	Outcomes	Limitations and challeges	Future perspectives
Genetic reporters for podocyte differentiation and toxicity Sharmin et al., 2016; Borestrom et al., 2018; Hale et al., 2018; Vanslambrouck et al., 2019; Yoshimura et al., 2019	The use of genome editing made it possible to knock in fluorescent proteins in the NPHS1 and MAFB loci. Their expression during podocyte differentiation in organoids was used to improve differentiation conditions and to establish a proof of concept for doxorubicin and PAN nephrotoxicity studies.	These studies did not carry out functional assays in podocytes following PAN and doxorubicin treatment.	These reporter cell lines could be shared through an international repository to accelerate the use of organoids in toxicology and drug screening. More sensitive reporters of podocyte damage could be developed with CRISPR/Cas9, to mark, for example, alterations on the localization of slit diaphragm proteins, and not only the total fluorescence. These lines could also be combined with microfluidic devices to generate more physiological models of podocyte and drug-induced toxicity.
Toxicity assays in organoid-derived tubular cells Freedman et al., 2015; Morizane et al., 2015; Takasato et al., 2015	In Morizane’s and Freedman’s work, KIM1 was upregulated in proximal and distal tubular cells of hiPSC-organoids in response to gentamycin and cisplatin treatment. KIM1 mRNA expression was upregulated in a dose-dependent manner. In Takasato’s work the activation of CASP3 was observed specifically in tubular cells following cisplatin exposure.	These organoid protocols have not been tested in a high-throughput toxicology setting. Additional iPSC lines and nephrotoxic agents and different iPSC lines are needed to validate the reproducibility of the protocols. Drug transporters will need to be analyzed (qualitatively and quantitatively) to assess similarities and differences with *in vivo* models.	To improve the applicability of organoids in toxicology, KIM1 reporter hiPSC lines could be generated to allow real-time observation of acute kidney injury and drug effect. Tubules assembled in microfluidic devices could also improve some structural functional features of tubular cells that are important for the toxic injury and repair response.

As with tubular cells hiPSC-derived podocytes in organoids are more similar to human podocytes than immortalized podocyte cell lines (Hale et al., 2018; Yoshimura et al., 2019). Gene expression analysis shows that immortalized cell lines lack important mature podocyte markers, have decreased functional capacities, such as albumin uptake, and decreased sensitivity to doxorubicin insult compared with hiPSC-derived podocytes (Musah et al., 2017; Rauch et al., 2018; Yoshimura et al., 2019). In addition, 3D culture of glomeruli-enriched organoids induces the expression of genes associated with slit diaphragm, renal filtration function and glomerular development compared with differentiated immortalized cell lines (Hale et al., 2018). These features make organoids potential candidates for podocyte toxicity studies and functional assays. To this end, the Little group knocked in a fluorescent reporter *BFP2* under the control of the podocyte marker *MAFB* in hiPSCs to generate glomeruli-enriched organoids, which were used to establish a model of doxorubicin-induced toxicity – a classical nephrotoxic drug that causes glomerular damage *in vivo* (Hale et al., 2018) – that allowed for fluorescence-based evaluation of toxicity in a dose-dependent manner.

In a similar approach Nishinakamura group (Yoshimura et al., 2019), used a NPHS1-GFP reporter cell line to optimize the differentiation conditions toward podocyte organoids and to perform toxicity studies. Puromycin aminonucleoside (PAN)-treated podocytes exhibited a significant and specific reduction of slit diaphragm proteins NEPH1 and Podocin at sub-lethal doses, while other podocyte markers remained unchanged, showing that organoid can be used to evaluate podocyte-specific drug toxicity.

In summary, the above studies highlight the potential of kidney organoids for toxicological screening of both tubular and glomerular structures. It is important to mention that 9% of safety failures of new drugs can be attributed to kidney toxicity in humans (Cook et al., 2014). Using human organoids for preclinical toxicity studies may increase the likelihood of candidate drugs succeeding in the clinical setting and at the same time reduce the costs of drug development.

Nonetheless, in order to fully exploit the organoid system in toxicity screens, it is necessary to generate large panels of organoids in an automated and miniaturized format. In a model of PKD, Czerniecki et al. (2018) provided proof-of-concept of how an organoid disease model can be adapted for drug screening. However, when miniaturizing their cultures, they observed a decrease in cystogenesis (20–40%) compared with their previous large-scale culture conditions (75%), highlighting the challenges of adapting organoid models into efficient drug screening formats. A summary of the different organoid toxicity assays is presented in Table 3.

## The Weaknesses of Kidney Organoids

Although organoids have significant advantages compared with classical cultures, they still have crucial insufficiencies when compared to the original organs (Xinaris, 2019). Organoids lack vasculature. This means that they are limited in terms of how much they can grow without cell death. However, following transplantation under the mouse kidney capsule, hiPSC-derived organoids have shown signs of growth, integration with the host circulatory system and further maturation, including the formation of the glomerular basement membrane (GBM), and maturation of the slit diaphragm in podocytes and brush border in tubular cells (Van Den Berg et al., 2018). Notably, hiPSC-derived organoids do not mature as well as transplanted embryonic kidney primordia (Rogers and Hammerman, 2004; Xinaris et al., 2012, 2016; Imberti et al., 2015). In addition, organoids also lack immune cells and therefore cannot be used to study processes that require this key component of human physiology, such as the inflammatory responses accompanying many nephropathies.

In addition, there are important defects in the cell composition of lab-grown organoids compared to the original organs. For example, in the analysis performed by Wu and collaborators using RNA sequencing, it was shown that organoids contain 10–20% off-target cells, such as neurons. The amount of each kidney cell type significantly varies according to the cell line and protocol that were pursued to generate organoids (Wu et al., 2018). According to Wu’s findings, the podocyte cluster was approximately 4% following Takasato’s differentiation protocol (Takasato et al., 2015), while 28% of cells were podocytes when Morizane’s was used (Morizane et al., 2015). Therefore, the efficiency and reproducibility of the differentiation protocol to produce specific cell types such as podocytes is an important aspect to consider when modeling human disease.

Another important limitation of kidney organoids is the limited ability to grow and mature *in vitro*. Several studies using single-cell RNA sequencing and immunofluorescence of kidney markers have shown that organoids do not mature further than an embryonic kidney does during the first trimester, even if maintained for long periods in culture (Takasato et al., 2015; Kim et al., 2017; Wu et al., 2018). This could be a limitation when aiming to model adult-onset diseases with organoids.

As existing culturing methods cannot faithfully replicate *in vivo* organogenesis conditions, organoids display important anatomic and structural insufficiencies. Organoids floating in media or embedded in artificial matrices *in vitro* lack the normal directional cues (both biochemical and mechanical) that drive the correct organization of cells within the organ. As a result, when kidney organoids self-assemble, tissues are developed somewhat randomly throughout the organoids. When the ureteric bud (UB; the precursor of the kidney’s collecting duct system) is absent or has developed randomly, the cortical-medullary differences in tissue organization that would normally be imposed by such a tree are missing. Anatomic malformations observed in human pluripotent stem cell-derived organoids, such as nephron-nephron connections and multi-branched nephrons may, in fact, be associated with this deficiency.

Important steps to generate more physiological kidney organoids have recently been taken, by differentiating UB progenitors and metanephric mesenchyme (MM; which gives origin to glomeruli and tubules) simultaneously or assembled in the same cell aggregate (Takasato et al., 2015; Taguchi and Nishinakamura, 2017). Since both populations of progenitors are derived from the intermediate mesoderm, Takasato et al. (2015) developed a differentiation protocol in which both UB and MM are generated in the same culture by varying the length of exposure to the Wnt agonist CHIR99021 and FGF9. They showed that these two populations gave rise to highly complex organoid structures composed of collecting ducts (GATA3+), nephron segments (podocytes, distal and proximal tubular cells), as well as endothelial and interstitial cells (Takasato et al., 2014, 2015). However, in later studies, Wu and collaborators (Wu et al., 2018) raised doubts about the presence of UB in these organoids. Based on a comparison of GATA3 clusters with adult kidney cell types, Wu concluded that GATA3 cells in Takasato’s organoids are metanephric mesenchyme-derived distal tubular cells rather than UB progenitors. More recently, Nishinakamura’s team took an important step toward solving the “UB hard problem” (Taguchi and Nishinakamura, 2017). The authors integrated UB and MM-derived cells into a 3D culture system to construct a quasi-physiological kidney organoid. Remarkably, branching morphogenesis of ureteric bud progenitors was observed and, when combined with nephron progenitor cells (NPCs), those cluster around the tips of the developing collecting-tree. Nonetheless, the branching morphogenesis of the collecting tree was more elaborated using mouse embryonic stem cells (mESC)-derived cells than in hiPSCs, stressing the differences between species and the need for further optimization when adapting the culture conditions from one cell type to the other (Taguchi and Nishinakamura, 2017).

Finally, hiPSC-derived organoids display commensurately high variability. Variability in organoids exists at many levels—between different starting cell lines or clones, between different genotypes, between batches of organoids, or even between areas of the same organoid itself (because of different local mircro-environmnets). Evaluation of the variability of organoids made by Takasato’s protocol showed that, although individual organoids are transcriptionally correlated, there is a significant variation between experimental batches, particularly in genes associated with temporal maturation (Phipson et al., 2019).

The variability across different cell lines or across different organoid preparations plagues the development of the organoid field because it limits its potential to incorporate other technologies, including computational science and bioengineering, which are required for developing high-throughput systems and mathematical models that can be used for phenotypic, toxicological and drug screens. Protocols for generating organoids are based on the knowledge of kidney development, but the accurate timing and amount of the different signaling molecules that are required for correct organogenesis remain mainly empirical. In order to better understand the *in vitro* development of organoids and to improve differentiation conditions, different research teams have generated reporter cell lines to observe in real-time the activation of specific developmental programs and renal cell markers (Kim et al., 2017; Vanslambrouck et al., 2019; Yoshimura et al., 2019). Using these tools, we could control the differentiation process by providing the appropriate spatiotemporal cues necessary for the efficient induction of specific cell types, tissue organization and maturation. This work can be further facilitated by CRISPR/Cas9 gene editing technology. Genome engineering is likely to be very useful for demystifying key mechanisms of human kidney organogenesis, as it will allow the generation of knockout lines, correct mutations in patient-derived hiPSCs, and a multitude of gene expression reporters. In addition, the use of CRISPR/Cas9 can facilitate the generation of reporter hiPSC lines (Sharmin et al., 2016; Borestrom et al., 2018; Howden et al., 2019; Vanslambrouck et al., 2019), which could be used to study developmental mechanisms, quantitatively the nephrotoxicity, de-differentiation of renal cells, fibrosis and other injury-associated molecular changes.

## Key Technical Milestones to Be Achieved

This review so far highlighted the advantages of kidney organoids for disease modeling and drug discovery, but also the challenges that must be addressed. We will now discuss a set of key technical milestones that in our opinion can help to circumvent the existing limitations, and strengthen organoids’ potential in biomedical research.

The first will be the generation of more hiPSC lines covering a wide range of kidney diseases, from genetic to sporadic diseases, postnatal to late onset diseases. Increasing the repertoire of disease-specific organoids will allow us to understand the role of the genotype in the disease process, and to uncover whether common or divergent disease mechanisms exist in patients with the same diagnosis and different genetic background. Moreover, by integrating high content analysis technologies such as RNA-seq and proteomics into organoid technologies we could identify the molecular networks that are altered in disease and govern organogenesis.

The implementation of microfluidic devices and micro-fabrication are a technological milestone that will help in the maturation of the organoid system. Homan et al., for instance, have shown that growing organoids in microfluidic devices increases the size of kidney organoids compared to static culture conditions (Homan et al., 2019). Chips designed to emulate tubular absorption *in vitro* have also been engineered (Weber et al., 2016; Vedula et al., 2017); these chips can be very useful for studying tubulogenesis and tubular function and testing drugs. Similar devices have recently been constructed to replicate glomerular filtration using different types of podocytes and endothelial cells (Petrosyan et al., 2019). If these technological improvements are combined with the advances made in the generation of podocyte-enriched organoids (Hale et al., 2018; Yoshimura et al., 2019), more physiological models of nephrons could be established on a larger scale for drug screening.

Bioengineering and 3D bioprinting approaches can be used to guide engineered tissues to pattern, differentiate and morph into more realistic organoids. Moreover, bioengineering the structural and physiological features that are necessary for modeling specific aspects of the organ separately can significantly minimize the inherent variability of self-organizing systems and facilitate the development of robust human models for comparative and quantitative drug testing studies. Indeed, three-dimensional printed polydimethylsiloxane (PDMS) scaffolds have been successfully used to grow complex kidney tubules with predefined architectures with remarkable reproducibility (Benedetti et al., 2018). This system was used to engineer patient-specific tubules, to model PKD and test drug efficacy, and to identify new therapeutic compounds for PKD. Moreover, this system has been applied to construct UB-like tubules from healthy individuals and a patient with a *PAX2* mutation and to study normal UB developmental processes and patient-specific defects. A different approach has shown that the local application of signals (e.g., bead-releasing morphogenetic factors) (Mills et al., 2017) or technical manipulations of engineered tissues (e.g., assembly of previously engineered organ component tissues) (Taguchi and Nishinakamura, 2017) could be used to add key missing information to self-organizing tissue to produce more anatomically realistic organoids.

In summary, the technological advances taking place in the organoid field, such as the generation of gene expression reporters, the increasing number of patient-derived hiPSC lines, the construction of microdevices to better mimic kidney function, bioengineering methods, are very promising developments for the study of kidney diseases and drug screening.

## Author Contributions

All authors contributed equally to the design and writing of this work.

## Conflict of Interest

The authors declare that the research was conducted in the absence of any commercial or financial relationships that could be construed as a potential conflict of interest.
